# Editorial: Forest microbiome: dynamics and interactions in the anthropocene era

**DOI:** 10.3389/fmicb.2025.1729625

**Published:** 2025-11-21

**Authors:** Amrita Chakraborty, Amit Roy, Shulin He, Antonio Castellano-Hinojosa, Fred O. Asiegbu, Teresa Ann Coutinho

**Affiliations:** 1 Faculty of Forestry and Wood Sciences, Czech University of Life Sciences Prague, Prague, Czechia; 2 College of Life Sciences, Chongqing Normal University, Chongqing, China; 3 Department of Microbiology, University of Granada, Granada, Spain; 4 Faculty of Agriculture and Forestry, University of Helsinki, Helsinki, Finland; 5 Department of Biochemistry, Genetics and Microbiology, Centre for Microbial Ecology and Genomics, Pretoria, South Africa; 6 Forestry and Agricultural Biotechnology Institute, University of Pretoria, Pretoria, South Africa

**Keywords:** forest microbiome, insect symbiosis, forest soil, forest management, microbial toolbox, tree microbiome

## Introduction

Forests represent one of the most complex and biodiverse ecosystems on Earth, with intricate networks linking trees, vegetation strata, insects, microbial communities, and soil processes. These networks, sustained by feedback loops and finely tuned ecological balances, form the foundation of global biogeochemical cycles and biodiversity conservation. At the core of these dynamics lies the forest microbiome, including a vast, often invisible consortium of bacteria, fungi, archaea, and viruses that mediates nutrient turnover, supports tree health, and shapes interactions across trophic levels (Baldrian, 2017; Asiegbu and Kovalchuk, 2021).

Increasingly, forest microbiome research reveals that microbial communities associated with soils, roots, leaves, and even insects form tightly interlinked networks that mediate ecosystem processes and responses to environmental change. In the Anthropocene era, climate change, deforestation, land-use intensification, pollution, and biological invasions are altering the composition and function of forest biomes at unprecedented scales (Baldrian et al., 2023). Shifts in tree diversity, changes in vegetation cover, and disruptions in soil structure ripple through microbial communities, reconfiguring ecological interactions and challenging our capacity to predict ecosystem trajectories (Lladó et al., 2017). Understanding how these elements interact under multidimensional anthropogenic pressures is therefore crucial. The forest microbiome is a pivotal, yet often overlooked, component of ecosystem dynamics. Unraveling its contributions is essential for a complete understanding of how forests respond to anthropogenic pressures. Microbes can also be used as valuable tools for forest pest management (Gupta et al., 2023). Hence, the current Research Topic is devoted to highlighting the microbial dimension of forest ecosystems. This Research Topic explores how vegetation, host traits, soil conditions, nitrogen inputs, and biotic stressors shape the diversity, structure, and function of microbial communities across forest compartments (rhizosphere, phyllosphere, endosphere, soil, and insect-associated niches). By integrating studies across multiple forest compartments, from soils to canopies and from roots to insects, this Research Topic advances a holistic understanding of the microbiome as a central axis of forest ecology, offering insights into feedback that drives ecosystem stability or vulnerability under global change.

## Forest soil microbiomes: structure across space, and time

Forest soil microbiomes exhibit strong spatial and temporal structuring across vegetation strata, elevation, and forest types. Vegetation layers exert a strong control on microbial composition and function, thereby coupling aboveground heterogeneity to belowground processes, as synthesized by Gilliam. Along elevational gradients, shifts in temperature, moisture, and vegetation composition as captured by soil pH, thermal regimes, and transitions among plant communities, emerge as primary determinants of microbial diversity, as shown by Li, Gao, et al. for steep gradients in Southwest China. Elevation and seasonality also structure the diversity of rare bacterial taxa in subtropical mountain forests. However, abundant and rare communities exhibit broadly similar elevational responses across seasons; their diversity, still, follows distinct trajectories, underscoring the ecological importance of rare taxa (Wu et al.). Seasonal dynamics further govern microbial assembly and ecosystem functioning in plantation forests, highlighting management-relevant temporal windows (Wang et al.). Multiple other edaphic factors, including temperature and precipitation, can also influence the composition of forest soil and rhizospheric microbiomes (Chakraborty et al., 2023b; Zádrapová et al., 2024). Collectively, these findings reveal that forest soil microbiomes respond sensitively to both natural gradients and anthropogenic interventions and are pivotal to ecosystem recovery, nutrient cycling, and resilience. Hence, the forest microbiome is key to evidence-based forest management and conservation.

## Tree microbiome: compartmentalization, disease, and decomposition

Tree microbiomes are strongly compartmentalized. Aboveground habitats such as the phyllosphere, stem, and needles, host microbial assemblages with ecological roles that diverge markedly from those in the belowground rhizosphere, one of the most dynamic zones of plant–microbe interaction. Empirical evidence is robust: Enea et al. showed that the phyllosphere and rhizosphere of sugar maple (*Acer saccharum* Marshall) harbour distinct communities that shift with environmental conditions, while Luo et al. reported pronounced bacterial and fungal turnover across leaves, roots, rhizosphere, and bulk soil in boreal forests. Collectively, these studies suggest that trees recruit and filter microbes through compartment-specific processes that underpin nutrient acquisition, stress tolerance, and overall fitness traits, which may ultimately constrain or facilitate range expansion.

Interestingly, in Norway spruce, Meng et al. found that infection by *Heterobasidion annosum* disrupts the within-tree microbial balance, with asymptomatic individuals maintaining higher richness and evenness than diseased counterparts; analogous disease-associated shifts were detected across leaves, roots, and soils of oil palm affected by leaf-spot, as shown by Azeez et al. Detrital phases are also no exception: Pan et al. demonstrated niche-specific colonization of epiphytic and endophytic fungi in decomposing larch leaf litter, identifying core phyllospheric consortia that synergistically regulate carbon flux, accelerate cellulose degradation, and enrich phosphorus and potassium. By linking phyllosphere composition to decomposition kinetics and nutrient stoichiometry, this work sharpens forecasts of boreal carbon cycling and offers practical indicators for monitoring ecosystem stability under climate change. Hence, compartment-specific assembly is not an accident but a governing principle of tree–microbe symbioses.

## Tree traits, planting design, and nitrogen deposition as drivers of microbial assemblages

Tree diversity, genotype, developmental stage, and nutrient enrichment strongly structure rhizospheric soil microbial communities. Across contrasting systems, species identity consistently modulates both microbial assemblages and soil chemistry. For instance, Frene et al. demonstrated that different tree species shape belowground communities and edaphic properties in plantations of *Juglans nigra* and *Quercus rubra*. Mixed stands can further enhance soil quality and microbial diversity. Li, Xie, et al. reported higher nutrient availability and a richer rhizosphere microbiota in mixed plantings compared to monocultures of *Parashorea chinensi*s. Domestication status also matters: Yang et al. found pronounced differences in rhizospheric diversity and function between wild and cultivated *Glyptostrobus pensilis*, while Lv et al. demonstrated that tree genotype and growth stage, together with soil chemistry, drive microbiome variation in Camellia forests. Collectively, these studies highlight the importance of integrating tree-specific traits, stand composition, and evolutionary history into forestry and ecosystem management.

Nitrogen inputs emerge as additional, powerful filters on microbial assembly by reshaping forest soil chemistry and community composition. In subtropical Guangxi forests, Jiang et al. demonstrated that nitrogen additions altered pH, nutrient status, and bacterial communities, with implications for long-term carbon storage and ecosystem stability. Crucially, the magnitude and direction of these nitrogen effects depend on host identity: Hou et al. documented tree species–specific microbial responses to nitrogen deposition, highlighting interactive controls between host traits and nutrient enrichment on rhizosphere assembly.

## Forest pest–microbe symbioses

Forest insects harbor microbes that are central to forest health, and the evidence is now overwhelming: these symbioses simultaneously drive insect adaptation and shape tree outcomes, for better or worse. Consider the bark beetles, where the insect–microbe partnership is anything but incidental. Khara et al. reveal finely tuned bacterial associations in two pine bark beetles that shift in response to environmental, host, and life-stage factors, forming an adaptive mosaic that equips beetles to navigate heterogeneous forest conditions. Building on this ecological context, Baños-Quintana et al. show that the Eurasian spruce bark beetle (*Ips typographus*) actively engineers its nursery: adults not only shape the microbiomes of their offspring but also remodel the gallery environment itself, carving out microbial micro-niches that amplify colonization success and, ultimately, beetle impact. These findings corroborate some of the observations from other studies on the same beetle (Chakraborty et al., 2023a).

At a broader phylogenetic scale, Pineda-Mendoza et al. map gut fungal assemblages across 14 *Dendroctonus* species, uncovering a conserved core mycobiome whose functions, such as digestion, nutrient acquisition, and cues for host specialization, are foundational to beetle fitness. Analogous studies on multiple *Ips* bark beetle guts also revealed a conserved core microbiota with similar functional potential (Chakraborty et al., 2020a,b). A microbiome study from termites further underscores that symbiotic interactions with microbes in wood-feeding insects can reverberate beyond nutrition: Setia et al. characterize the bacterial consortia of *Coptotermes gestroi* on ironwood (*Casuarina equisetifolia*) and demonstrate how infestation physically and microbially primes tissues for pathogen ingress, accelerating disease trajectories and hastening decline.

Nevertheless, forest insects are ecosystem engineers because their microbiomes are ecosystem tools. These invisible microbial communities facilitate host adaptation, enhance digestion, and, in many systems, contribute to exacerbating tree mortality. The intricate microbial contribution also increases the complexity of the forest networks when pests adapt to numerous anthropogenic pressures. Furthermore, the adaptation of pests also poses a significant threat to the forest health under these pressures. Together, these findings highlight that insect–microbe interactions are not peripheral but central to forest health. Ignoring these dynamics risks undermining pest management and forest conservation strategies.

## Management and restoration: trajectories of microbial recovery

Forest management and restoration must be grounded in the trajectories of microbial recovery and the soil functions that underpin them. Drawing on logged Bornean lowland dipterocarp rainforest, Robinson et al. demonstrate that active restoration outpaces passive approaches in rebuilding belowground communities, resulting in higher microbial diversity and functionality across multiple spatial scales. The implication is uncompromising: restoration strategies that fail to target explicit recovery of soil microbiota will underperform in conserving biodiversity and re-establishing critical ecosystem processes. Complementing this view, Zhang et al. demonstrate that reforestation regimes shape soil microbial assemblages primarily by modifying root traits and soil physicochemical conditions, while soil metabolites contribute comparatively little. Taken together, these studies demand a tighter integration of soil metabolomics with microbial community analyses to elucidate how plant traits and soil chemistry drive microbial dynamics, and to sharpen predictive models guiding forest restoration under diverse reforestation regimes.

## Conclusion and perspectives

**Figure 1:**
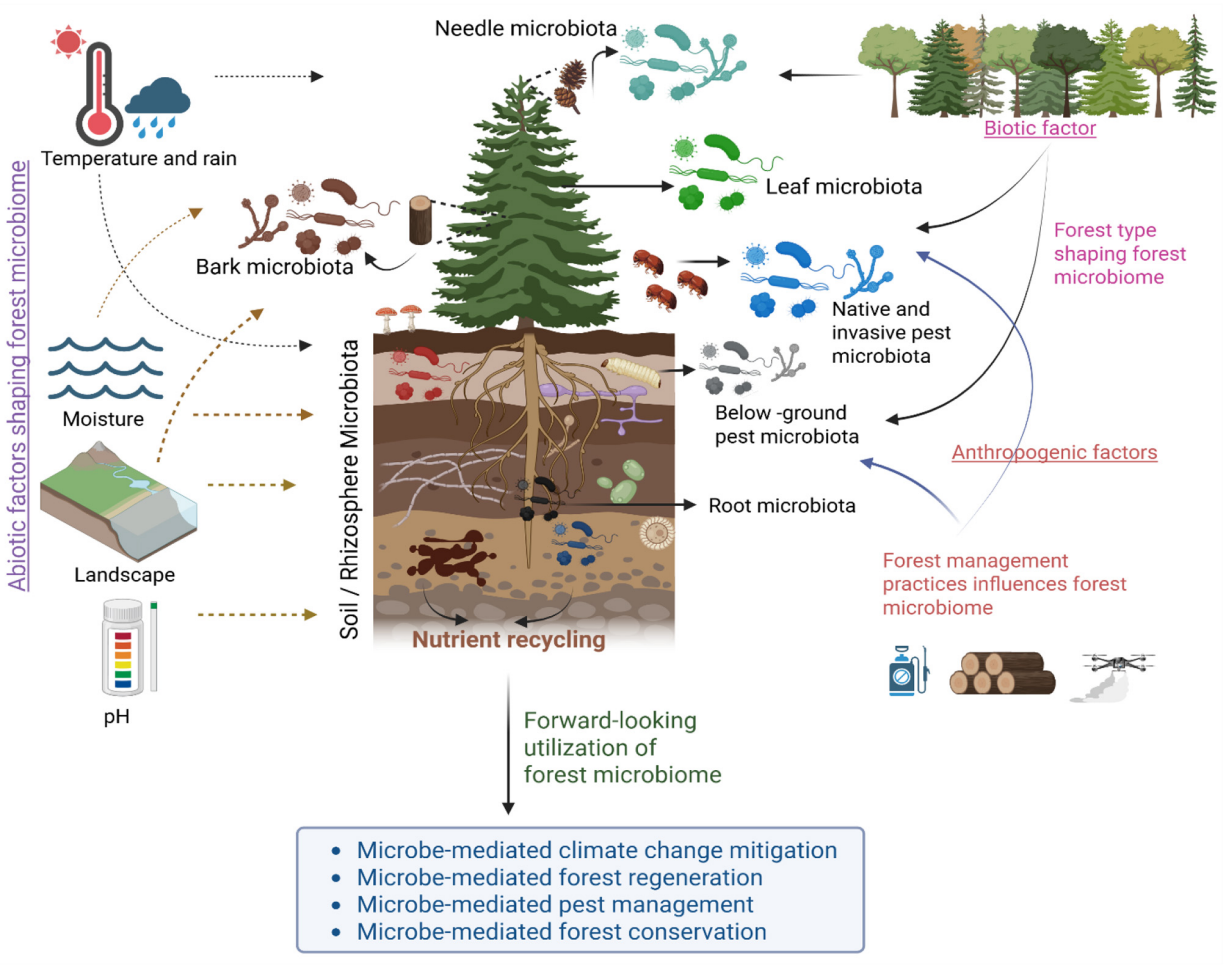
Conceptual framework of factors shaping the forest microbiome and its ecological implications. The forest microbiome is structured by multiple interacting abiotic, biotic, and anthropogenic factors. Abiotic drivers such as temperature, precipitation, moisture, landscape configuration, and soil pH influence the diversity and composition of microbial communities associated with various forest compartments, including the needle, leaf, bark, root, and soil (rhizosphere) microbiota. Biotic factors, including forest type and pest communities (both native and invasive), further shape microbiome structure and function. Anthropogenic factors, notably forest management practices, exert additional influences on microbial assemblages. The forest microbiome contributes to critical ecosystem processes, such as nutrient recycling, and presents forward-looking opportunities for sustainable forestry through microbe-mediated climate change mitigation, forest regeneration, pest management, and forest conservation. Figure created in Biorender.

Forest microbiome represents an integrative, multi-kingdom infrastructure that links soils, roots, leaves, and insects to regulate nutrient cycling, plant health, and ecosystem resilience under global change. Forest microbiomes cannot be understood in isolation, and they form a continuum across compartments that jointly sustain forest health and resilience. We propose a conceptual framework that illustrates how abiotic, biotic, and anthropogenic factors converge to shape the forest microbiome, which in turn drives nutrient recycling, forest regeneration, pest management, and climate change mitigation (Figure 1).

The studies featured in this Research Topic demonstrate that microbial diversity across soil, vegetation, and insect hosts is deeply embedded within the ecological fabric of forest ecosystems. They highlight how abiotic factors (e.g., temperature, precipitation, and soil pH), biotic interactions (e.g., host identity and pest communities), and anthropogenic pressures (e.g., nitrogen deposition and forest management) jointly shape microbial assembly and, ultimately, ecosystem trajectories.

Looking forward, advancing from taxonomic surveys toward functional approaches, such as metatranscriptomics, metabolomics, and targeted functional assays, will be essential to uncover the mechanistic links between microbiomes and ecosystem processes. Integrating multi-compartment studies that connect soil, rhizosphere, phyllosphere, and insect-associated microbiomes will enable a deeper understanding of how climate change, nutrient enrichment, and land-use intensification reshape forest stability. Furthermore, the integration of mutualistic desirable microbiomes is expected to be of great significance for sustainable forest production and translational forest management. Additionally, future research directions may explore the application of novel approaches, such as metagenome-wide association studies (MWAS), to link the relative abundance of specific genes in the metagenome with certain forest tree diseases.

This knowledge is not merely academic. It provides a foundation for microbiome-informed strategies in forest conservation, restoration, pest management, and climate change mitigation. Harnessing the power of microbial networks will be pivotal for designing resilient forest ecosystems in the Anthropocene.
